# Immediate and late effects of supraphysiological doses of nandrolone decanoate on the testis of rats

**DOI:** 10.1590/acb402925

**Published:** 2025-03-31

**Authors:** Bruno Felix-Patricio, Roger Gaspar Marchon, Bianca Martins Gregório, Diogo Benchimol De Souza

**Affiliations:** 1 Universidade Federal Fluminense – Department of Natural Sciences – Rio das Ostras (RJ) – Brazil.; 2 Universidade do Estado do Rio de Janeiro – Urogenital Research Unit – Rio de Janeiro (RJ) – Brazil.

**Keywords:** Anabolic Androgenic Steroids, Nandrolone Decanoate, Testis

## Abstract

**Purpose:**

To investigate the effects of supraphysiologic doses of nandrolone decanoate on the testicular morphology of rats and if these effects could be transitory or permanent.

**Methods:**

Twenty-five male rats were divided in four groups. C28 and C40 were control rats killed with 28 and 40 weeks old, respectively; and T28 and T40 were treated with nandrolone decanoate (10 mg/kg) and killed immediately after the treatment (T28) or 12 weeks after the end of treatment (T40). The testis weight and volume were measured, and the seminiferous tubule area and epithelium height were assessed by histomorphometric methods.

**Results:**

The seminiferous tubules area and epithelium height of group T28 were reduced in comparison to C28. Group T40 showed reduced testicular weight and volume, as well as seminiferous tubule area and epithelium height in comparison to C40.

**Conclusion:**

The use of nandrolone decanoate promotes major structural modifications on the testes of rats. These modifications are even worse 12 weeks after ending the use of the anabolic androgenic steroid.

## Introduction

Anabolic androgenic steroids (AAS) are important hormones used since 1930 for hypogonadism, osteoporosis, cachexia delayed puberty and breast cancer treatments^1^^,^ 2. However, in the present days, synthetic testosterone analogs have been indiscriminately used by professional and amateur athletes as an attempt to improve their athletic performance, appearance, and muscle mass^3^^,^ 4.

Besides adverse effects on different organs, which even result in a shorter life span for mice^5^, AAS are associated with morphological alterations of the urogenital organs. It was previously reported that AAS alters the proportions of corpus cavernosum tissues in both pubertal and adult rats, which may be associated with erectile dysfunction4. The prostate of animals receiving AAS also showed important structural alterations, with reduced weight and volume^6^^,^ 7.

Regarding the testicle, some studies pointed to a reduction of spermatozoid quality and fertility, both in humans and rats treated with AAS. This seems to be a result of the altered hypothalamic-pituitary-gonadal axis since these synthetic hormones downregulate the endogenous synthesis of testosterone^8^^–^ 10. However, few studies performed quantitative testicular histomorphometry to objectively evaluate the impact of AAS usage on the testis.

Further, it is unknown if the altered testicular morphology could be reversed after stopping AAS usage. Thus, the objective of this study was to investigate the effects of supraphysiologic doses of nandrolone decanoate on the testicular morphology of rats and if these effects were transitory or permanent.

## Methods

This study was conducted in the Laboratory of Research in Human and Experimental Biology, at the Universidade Federal Fluminense. All experiments were performed in accordance with the Brazilian laws for scientific use of animals, and the project was approved by the local ethics committee (protocol no. CEUA/755). Animals were kept in a room with controlled temperature (22 ± 2 °C) and an artificial dark-light cycle (lights on from 7 a.m. to 7 p.m.). The rats received standard rat chow and water *ad libitum*.

Twenty-five male Wistar rats, weighing 350 to 450 g of body mass, were used in this study. The animals were randomly divided into four groups:

- C28 (n = 5): control rats killed with 28 weeks old;
- T28 (n = 6): rats treated with AAS and killed immediately after the treatment;
- C40 (n = 7): control rats killed at 40 weeks old;
- T40 (n = 7): rats treated with AAS and killed 12 weeks after the end of the treatment.

Groups T28 and T40 received nandrolone decanoate (Deca Durabolin 50 mg·mL^-1^, Organon, São Paulo, SP, Brazil), at the dose of 10 mg·Kg^-1^ of body mass. Meanwhile, the control groups (C28 and C40) were injected with 90% peanut oil diluted in benzoic alcohol^4^^,^ 6. Both the steroid hormone and carrier were administered by intramuscular injection once a week for eight weeks (from 20^th^ until 28^th^ week old). This treatment protocol was established to simulate a commonly used protocol in humans, as previously published^4^^,^ 6. During the study, body weight was weekly measured. The rats were killed by anesthetic overdose with an intraperitoneal thiopental injection (Thiopentax, Cristália, Itapira, SP, Brazil) when they were 28 (C28 and T28) or 40 weeks old (C40 and T40).

The testis was dissected, weighed, and its volume measured by using Scherle’s method^11^. The organ was fixed by immersion in a 4% buffered formaldehyde solution for at least 24 hours. After this period, the testicle was transversally cut into 3-mm sections and processed for paraffin embedding. Five micrometer-thick sections were obtained. Morphometric analysis was performed on hematoxylin and eosin-stained slices, captured using an Olympus BX51 microscope with a coupled DP70 digital camera (Olympus, Tokyo, Japan). All morphometrical analyses were assessed with the ImageJ software (NIH, Bethesda, United States of America)^12^.

The seminiferous tubule area was evaluated with 25 images per animal, using images obtained at ×100 magnification. For this measurement, after calibration, the “free hand selection” tool was used to delineate each tubule^13^^–^ 15. The seminiferous epithelium height was also measured in 125 tubules per rat, using images obtained at ×200 magnification. For this analysis, three equidistant lines were drawn from the tunica propria of the seminiferous tubules to the last germinative cell, thus excluding the spermatozoa. The mean of these three lines was considered as the height of that seminiferous tubule^14^^–^ 17.

For each parameter, the mean of each control group was compared to its age-matched treated group. Results were first analyzed using the Kolmogorov-Smirnov’s normality test. Parametric data were then compared using the Student’s t test, while nonparametric data were compared using the Mann-Whitney’s test. For all analyses, two-tailed tests were used. All analyses were performed using the GraphPad Prism 5.0 software (GraphPad Software, San Diego, CA, United States of America). Mean differences were considered significant at *p* < 0.05. All results are presented as mean ± standard deviation.

## Results

The administration of the anabolic steroid did not alter the body mass among the groups (*p* = 0.52 for C28 vs. T28; *p* = 0.66 for C40 vs. T40). Similarly, the testicular weight and volume were similar among groups C28 and T28 (*p* = 0.23 and *p* = 0.28, respectively). However, group T40 showed a 49.55% reduced testicular weight (*p* < 0.0001), and 52.87% reduced testicular volume (*p* < 0.0001) in comparison to C40.

**Figure 1:**
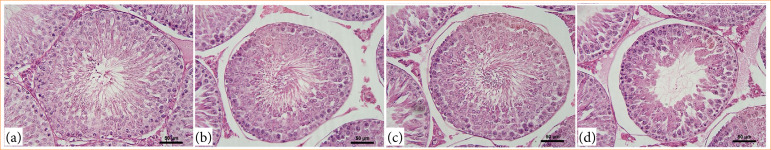
Photomicrographs showing seminiferous tubule of controls and rats treated with nandrolone decanoate. **(a)** Seminiferous tubule of group C28. **(b)** Seminiferous tubule of group T28. **(c)** Seminiferous tubule of group C40. **(d)** Seminiferous tubule of group T40. Hematoxylin and eosin 400X.

Source: Elaborated by the authors.

**Figure 2:**
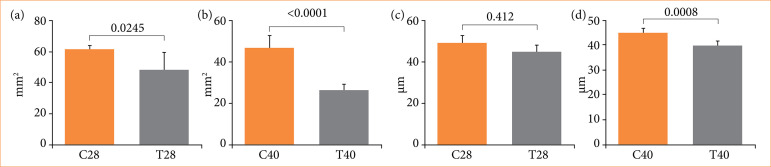
Comparative graphics with morphometric results of controls and rats treated with nandrolone decanoate. **(a)** Seminiferous tubule area of groups C28 and T28. **(b)** Seminiferous tubule area of groups C40 and T40. **(c)** Seminiferous epithelium height of groups C28 and T28. **(d)** Seminiferous epithelium height of groups C40 and T40. Data expressed as mean and standard deviation.

Source: Elaborated by the authors.

Regarding the histomorphometrical testicular analysis, differences were observed in groups C28 and T28. The seminiferous tubule area of group T28 was reduced by 21.86% (p = 0.0245), and the seminiferous epithelium height was reduced by 9.33% (*p* = 0.0412) in comparison to C28. Animals in group T40 showed a 43.89% reduction in the seminiferous tubule area (*p* < 0.0001) in comparison to C40, and a 11.99% reduction in seminiferous epithelium height (*p* = 0.0008) in comparison to C40, as represented in Figs. 1 and 2. All data are presented in Table 1.

**Table 1:** Morphological data of rats treated with nandrolone decanoate for eight weeks compared with age-matched controls immediately after the end of treatment (C28 and T28) or after 12 weeks of the end of treatment (C40 and T40)*.

	C28	T28	p-value	C40	T40	*p*-value
Body weight (g)	366.3 ± 22.3	386.0 ± 19.2	0.5186	405.0 ± 19.5	414.3 ± 9.1	0.6588
Testicular weight (g)	1.62 ± 0.09	1.48 ± 0.05	0.2299	1.82 ± 0.03	0.91 ± 0.07	< 0.0001
Testicular volume (mL)	1.54 ± 0.08	1.42 ± 0.07	0.2782	1.73 ± 0.03	0.82 ± 0.07	< 0.0001
Seminiferous tubules area (mm^2^)	61.87 ± 0.99	48.34 ± 4.20	0.0245	46.68 ± 2.62	26.19 ± 1.14	< 0.0001
Seminiferous epithelium height (µm)	49.39 ± 1.52	44.78 ± 1.26	0.0412	44.93 ± 0.82	39.54 ± 1.79	0.0008

* Data are presented as mean ± standard deviation.

Source: Elaborated by the authors.

## Discussion

AAS are synthetic derivatives of testosterone that have anabolic and androgenic characteristics and are capable of promoting both the development of secondary sexual characteristics and an increase in muscle strength and mass, improving physical fitness^18^^,^ 19. In contrast, many studies show that the use of AAS can lead to important structural changes in some organs of the male genital system, such as the prostate^6^, the penis^4^, and the testicles^20^^–^ 22. Our study showed that immediate and delayed administration of nandrolone decanoate did not alter the biometric parameters (body mass and food intake) of the animals. However, the morphology of the testicle was modified, which can directly compromise fertility.

Some authors reported that the administration of nandrolone decanoate is related to the retention of fluids and sodium in the body, which can generate an increase in body mass^23^. In the present study, the animals treated with AAS (groups T28 and T40) had the same body mass as their respective controls, which corroborates other studies^22^^,^ 24. Furthermore, food intake did not differ between the different groups studied, which justifies the similar weight of the animals.

Regarding the histomorphometric parameters, our data showed that delayed administration of nandrolone decanoate reduced testicular weight and volume. These changes are not observed immediately after the end of the treatment, but only 12 weeks later. Shokri et al.^22^ showed that a supraphysiological dose of nandrolone decanoate reduced these same parameters. It is already well established in the literature that the reduction in testicular volume is directly related to organ atrophy. Although this study was exclusively morphological and did not perform any hormonal measurement, it is known that high doses of compounds derived from testosterone can act on the hypothalamic-pituitary-gonadal axis, interfering with the secretion of hormones such as follicle-stimulating hormone and luteinizing hormone. As a result, the supporting role of Sertoli cells may be lost and spermatogenesis impaired^25^. Still regarding the fertility, it is known that the mitochondrial membrane of the testicles is rich in polyunsaturated lipids, which may make them vulnerable to peroxidative injury^26^. Chaves et al.^27^ indicated that chronic treatment with AAS negatively interferes with the activity of antioxidant enzymes, which may induce oxidative stress and culminate in disturbances in the spermatogenic cascade and azoospermia.

Additionally, the area of the seminiferous tubule, a parameter widely used to assess spermatogenesis, was smaller in both T28 and T40 groups. Although the immediately analyzed group did not show macroscopic changes in the testis, the reduction of the area of the seminiferous tubule may indicate deficient sperm production^15^^,^ 28. Likewise, the analyses 12 weeks after the end of the treatment of the animals maintained the reduction in this parameter, indicating that even after stopping the treatment there is no reversal of this morphological change. Regarding the height of the seminiferous epithelium, we found that the use of supraphysiological doses of nandrolone decanoate was also harmful to the animals both when evaluated immediately after the end of the treatment and when evaluated 12 weeks after the treatment. It was observed that the morphology of the organ was highly compromised by the steroid treatment, which may compromise sperm production. These modifications are not reverted after stopping the use of AAS. On the contrary, in a general manner, it seems to be worse 12 weeks after the end of the treatment.

Considering the above, it was found that treatments with high doses of nandrolone decanoate promoted significant microscopic and macroscopic changes in the testicles of adult rats, with a reduction in the area and height of the seminiferous tubule epithelium, and in the weight and volume of the organ. Further studies involving hormonal measurements are necessary to justify and correlate all the structural changes found.

The study has some limitations that should be pointed. Although rodents are widely used, the results of animal studies should not be directly transduced to humans. Future clinical studies focusing on the impact of AAS on testicular function, both immediately and some weeks after the end of the treatment, are warranted. Furthermore, studies with longer follow-up periods after the treatment are required to better understand the long-term effects of AAS on testes.

## Conclusion

The use of supraphysiological doses of nandrolone decanoate promotes major structural modifications on the testes of rats. The evaluation 12 weeks after the end of AAS use showed even worse results, including testicular atrophy. These modifications can be related with fertility impairment.

## Data Availability

All data sets were generated or analyzed in the current study.
